# N-Acetyltyrosine as a Biomarker of Parenteral Nutrition Administration in First-Tier Newborn Screening Assays

**DOI:** 10.3390/ijns10040081

**Published:** 2024-12-10

**Authors:** C. Austin Pickens, Samyukta Sah, Rahul Chandrappa, Samantha L. Isenberg, Elya R. Courtney, Timothy Lim, Donald H. Chace, Rachel Lee, Carla Cuthbert, Konstantinos Petritis

**Affiliations:** 1 Division of Laboratory Sciences, National Center for Environmental Health, Centers for Disease Control and Prevention, 4770 Buford Hwy NE, S110-3, Atlanta, GA 30341, USA; cpickens@cdc.gov (C.A.P.); jyw9@cdc.gov (S.S.); sisenberg@cdc.gov (S.L.I.); ecourtney2@cdc.gov (E.R.C.); thl5@cdc.gov (T.L.); nmo1@cdc.gov (R.L.); ijz6@cdc.gov (C.C.); 2 Capitainer Inc., East Providence, RI 02914, USA; donald.chace@capitainer.com

**Keywords:** newborn screening, parenteral nutrition, metabolic disorders, multiplexing

## Abstract

Parenteral nutrition (PN) is a nutrient solution administered intravenously (IV) to premature babies. PN causes elevations of some amino acids in blood samples that are also biomarkers used in newborn screening (NBS). Therefore, PN status must be annotated by clinicians on dried blood spot (DBS) cards to reduce NBS laboratory burdens associated with potential false results; however, NBS laboratories continue to receive DBSs with misannotated PN status. N-acetyltyrosine (NAT), a water-soluble tyrosine analog used to increase tyrosine bioavailability in PN solutions, can be used as a blood-based biomarker of PN administration in NBS assays. Residual DBS specimens and manufactured DBSs were used in analyses. The assay was developed and validated using flow injection analysis tandem mass spectrometry (FIA-MS/MS) for the detection of NAT. NAT was only present in neonate DBSs with annotated PN administration and was multiplexed into first-tier newborn screening assays. NAT was highly correlated with amino acids present in PN solutions, such as arginine, leucine, methionine, phenylalanine, and valine. In our sample cohort, we determined an NAT cutoff could aid the identification of misannotated neonates administered PN. We also report the Amadori rearrangement product valine–hexose (Val-Hex) was quantifiable in neonates administered PN, which we suspect forms in the PN solution and/or IV lines. Here, we present the first known use of NAT as a biomarker of PN administration, which is currently being piloted by two U.S. NBS laboratories. NAT and Val-Hex can aid the identification of misannotated DBSs from neonates administered PN, thus decreasing false positive rates.

## 1. Introduction

Parenteral nutrition (PN) is a solution of essential nutrients administered intravenously (IV) to treat malnutrition [1], most commonly during the first days of life in very premature low-birthweight infants. It is sometimes referred to as total PN (TPN). PN is prepared in the hospital pharmacy, where concentrated macronutrients, including amino acids and lipids, and micronutrients, such as vitamins and minerals, are diluted to various concentrations in a 5 or 10% dextrose solution [2,3]. Newborns receive PN for various reasons, including premature birth, necrotizing enterocolitis [4], and other intestinal disorders [5]. Premature birth is a nutritional emergency [5], and PN is often administered upon admission to the neonatal intensive care unit (NICU) to ensure adequate protein intake [6]. It is estimated 9–13% of neonates are admitted to an NICU [7], and up to 70% of NICU neonates are prescribed PN [8]. It is important to note that PN contains free amino acids, not intact proteins, and the protein intakes of premature babies are paired with appropriate gestational ages. For instance, premature babies of 24 to 30 weeks have protein requirements ranging from 3.5 to 4.0 g/kg/day, while premature babies of 34 to 36 weeks range from 2.5 to 3.0 g/kg/day [6].

There are still reports of postnatal growth failure in premature neonates despite numerous efforts to provide adequate PN in NICUs [9,10]. The postnatal growth failure in premature neonates administered PN may be associated with an incomplete understanding of nutrition and metabolism, i.e., the immature metabolic pathways [11,12] in low-birthweight NICU infants. For instance, one study administered adult PN solutions with higher levels of phenylalanine (Phe), aiming to deliver higher tyrosine (Tyr) by relying on enzymatic conversion by Phe hydroxylase [13]. However, in neonates, the high Phe appeared to overload the Phe metabolism, causing elevated Phe and Phe catabolite excretion rather than shunting completely to Tyr biosynthesis [13]. Tyr is an aromatic amino acid with low solubility in water, which limits Tyr to < 1% of total amino acids in PN solutions, while aromatic amino acid requirements in neonates were estimated at 3.1% to 3.9% of the total [14]. It was hypothesized that Tyr deficiency in PN solutions could trigger low thyroxine since both thyroxine and Tyr were similarly low [15] in a PN randomized clinical trial. The rationale was that low Tyr availability in PN could impact thyroxin biosynthesis since the protein thyroglobulin is essential for thyroxin synthesis, and thyroglobulin contains roughly 140 Tyr residues, with roughly 30 being iodinated [16].

In neonatal PN solutions, Tyr solubility limitations are overcome by adding an acetylated form of Tyr known as N-acetyltyrosine (NAT), along with Tyr. NAT is found in PN solutions prescribed to NICU neonates in the U.S. [17,18], and it is not present in adult PN solutions. Nearly 4 decades ago, NAT was described as a higher soluble form of Tyr for PN, increasing Tyr incorporation into tissues after NAT IV infusion [19]. However, NAT is poorly metabolized to Tyr by hepatic and renal enzymes [20], and although NAT administered by PN does increase Tyr in tissues, a large proportion of intact NAT is present in blood and excreted in urine [21,22]. Poor NAT metabolism appears to be a phenomenon unique to neonates and children younger than twelve months of age compared to children aged one to seven years and may be related to the oversaturation of deacetylases and immature metabolism of neonates [23].

Newborn screening (NBS) analyzes biomarkers from dried blood spots (DBSs) collected shortly after birth to identify pre-symptomatic newborns at risk of rare inborn errors of metabolism (IEMs). Flow injection analysis–tandem mass spectrometry (FIA-MS/MS) is used to screen the majority of NBS biomarkers and diseases included in the U.S. Recommended Uniform Screening Panel [24]. Many NBS amino acid biomarkers elevated in the presence of IEMs are the same amino acids present in PN solutions. In 2007, an expanded NBS program in the Netherlands documented 14 false positive cases of homocystinuria over two months, all originating from a single NICU that was using PN solutions with a high methionine (Met) content [25]. Since PN elevates levels of amino acids and other biomarkers in the blood, clinicians annotate PN status on DBS cards after collection, so NBS laboratories anticipate elevated biomarkers and report inconclusive screening results for selected IEMs. However, if the PN status is not correctly annotated, the neonate can be identified as a presumptive positive for one or more IEMs. This occurrence in NBS laboratories is burdensome because both running additional tests and/or reaching out to hospital staff to confirm the PN status is time-consuming [26]. Also, the false positive results from a misannotated PN specimen could lead to a parent being notified that their premature newborn is also at risk for an IEM, which increases parental anxiety [27]. Since NAT is uniquely present in neonatal PN solutions, poorly metabolized, and detectable in blood, we hypothesized that NAT could be used as a DBS-based biomarker of PN administration in NBS assays.

## 2. Materials and Methods

### 2.1. Sample Type and Preparation

Detailed information on clinical specimens, quality control (QC), and linearity materials used in analyses; method optimization; extraction methods; solvents and chemicals; and unlabeled biomarkers and isotopically-labeled internal standards (ISs) are provided in Supplementary Materials. Certified values for linearity materials and QCs are presented in Tables S1 and S2.

### 2.2. Acquisition, Quantification, and Data Analysis

Electrospray ionization source parameters, peak integrations, and quantification methods are presented in Supplementary Materials. The biomarker parent and product ion *m*/*z*, the IS employed for quantification, and the cone and collision voltages are presented in Table S3. All quantified data and peak area data were exported as spreadsheets and imported into R, version 4.2.1 [28], for analysis and visualization.

## 3. Results

**Figure 1:**
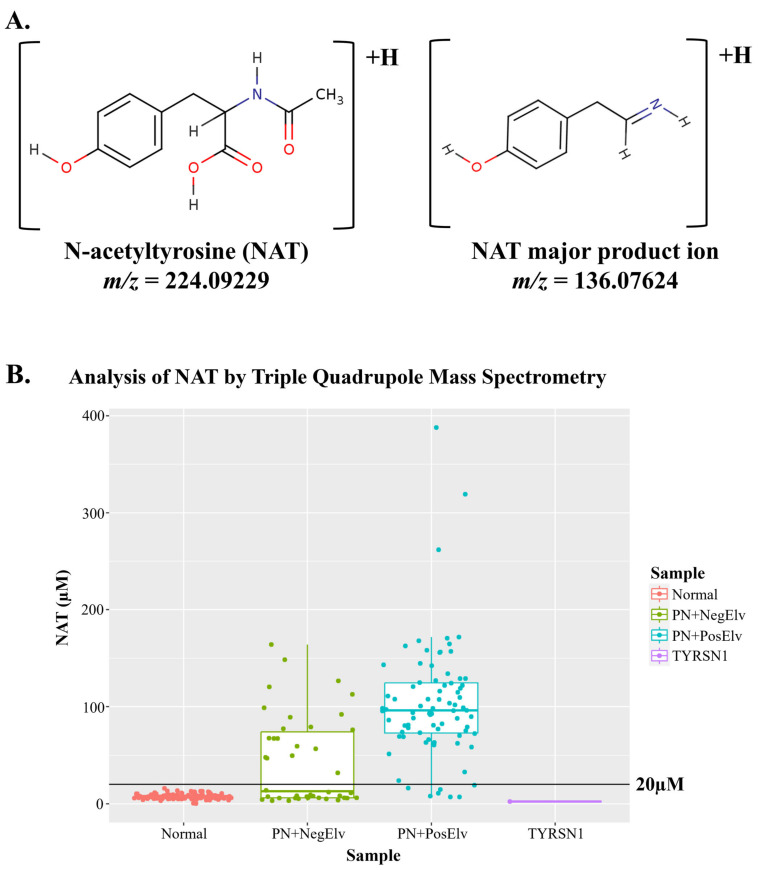

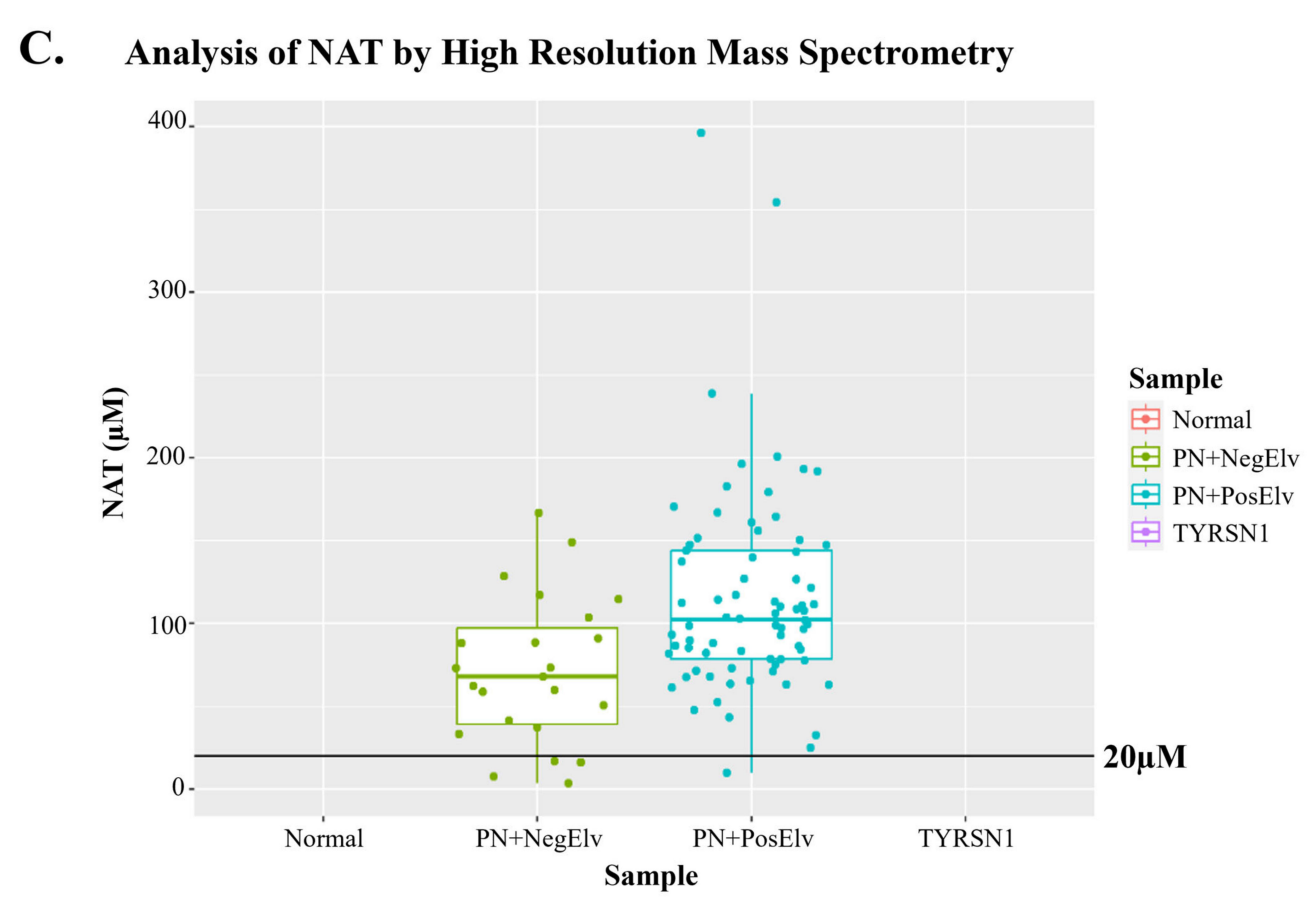
N-acetyltyrosine (NAT)structure and distribution among residual DBS clinical samples. Analysis of NAT in residual clinical samples was performed by flow injection analysis–tandem mass spectrometry. (**A**) displays the structure of NAT and major product ion, along with the accurate M+H *m*/*z*, respectively. (**B**,**C**) display boxplots of NAT concentrations in residual clinical specimens, where the solid black horizontal line denotes an NAT concentration of 20 µM. Data in (**B**) were acquired using a triple quadrupole mass spectrometer, while data in (**C**) were acquired using a high-resolution mass spectrometer. In (**C**), since the accurate *m*/*z* of NAT’s product ion was not present in any non-PN+ specimens, the data are NA instead of zero, which explains the absence of data in (**C**) when compared to (**B**). PN+: administered total parenteral nutrition; Normal: presumptive normals (*n* = 120); PN+NegElv: PN+ with no elevated biomarkers (*n* = 42); PN+PosElv: PN+ with one or more elevated biomarkers (*n* = 80); TYRSN1: neonate with tyrosinemia type I (TYRSN1, OMIM 276700, *n* = 1).

The chemical structure of NAT, its product ion, and corresponding accurate M+H *m*/*z*s are presented in Figure 1A. Analysis of the residual NBS samples by FIA-MS/MS on a triple quadrupole platform appears to indicate NAT was present in all samples (Figure 1B). This was surprising; however, using a 20 µM NAT cutoff, we could correctly identify 91.25% of neonates with reported PN administered (PN+), with one or more elevated biomarker (PN+PosElv) samples (i.e., 73 of 80), with no presumptive normals being misidentified. Based on our cohort, if their DBS cards were not correctly annotated as PN+, a significant number of potential IEM false positives (i.e., PN+PosElvs) could be flagged as PN+ solely using NAT.

The presence of NAT in all residual NBS specimens was unusual given that NAT is not reported as an endogenous blood-based metabolite; therefore, we suspected an interference in the *m*/*z* 224.1 > 136.1 NAT transition in the triple quadrupole platforms. The residual NBS specimens were re-analyzed by FIA coupled to high-resolution mass spectrometry (HRMS) using parallel reaction monitoring, and we determined an *m*/*z* of 136.0870 present in the NAT *m*/*z* 224.1 > 136.1 transition integrated in the triple quadrupole platforms (Figure S1A,B). Figure 1C clearly displays NAT was only present in PN+ specimens when analyzed by HRMS (i.e., 74 of 80 PN+PosElv specimens, 92.5%), and six of the seven PN+PosElv specimens that had NAT < 20 µM (Figure 1B) did not have any detectable NAT present in the blood (Figure 1C). We hypothesized that these six PN+PosElv neonates had been taken off PN [26] and/or transitioned to enteral feeding, and their DBS cards were still annotated as PN+; thus, there was sufficient time for NAT urinary excretion prior to their DBS collection. It is important to note NAT was found in the urine of tyrosinemia type 1 (TYRSN1, OMIM 276700), so we included a TYRSN1 (*n* = 1) confirmed case and, as expected, found NAT was not detectable in their DBS.

**Figure 2:**
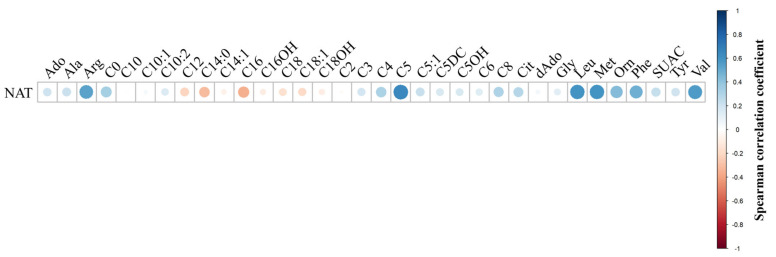
Bubble correlogram of Spearman correlation coefficients of neonates administered parenteral nutrition. N-acetyltyrosine (NAT) concentration data were correlated with other biomarkers in the assay, using only data from neonates with reported parenteral nutrition administration (PN+, *n* = 122). Spearman correlations were performed, and coefficients were plotted using a bubble correlogram, where the size and color of the bubble denote the direction of correlation and proximity to 1 or −1. NAT was positively correlated with amino acids present in parenteral nutrition solutions. The acylcarnitine C5 was positively correlated with NAT since C5 is documented to be elevated in PN-administered neonates.

Next, concentrations of biomarkers in the PN+ specimens were correlated using Spearman correlations, and a bubble correlogram of Spearman coefficients was generated. The full bubble correlogram is presented in Figure S2. Figure 2 displays that NAT was highly correlated with arginine (Arg), leucine (Leu), Met, Phe, and valine (Val). This was expected since NAT is present in PN solutions with these amino acids, so an increase in NAT should result in a concomitant increase in amino acids. Isovalerylcarnitine (C5) was positively correlated with NAT, which is not present in PN solutions; however, it is well-known that C5 is elevated in PN+ neonates as a metabolic byproduct [29].

**Figure 3:**
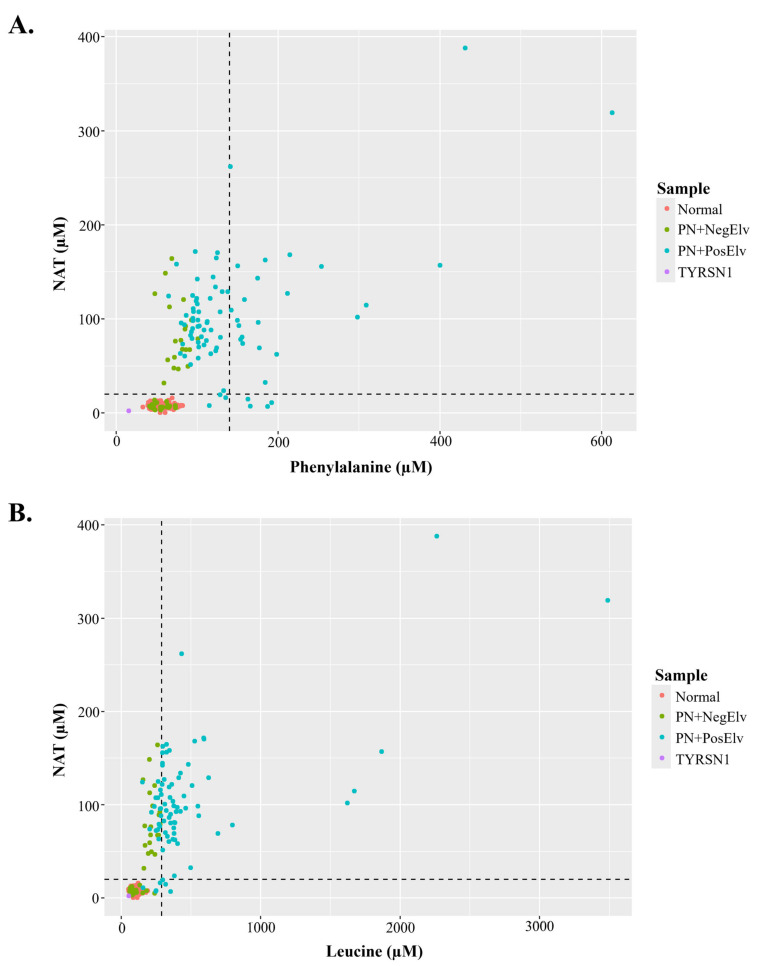
N-acetyltyrosine (NAT) bi-plots. (**A**,**B**) display bi-plots of NAT with an amino acid. In each plot, sample groups are denoted by color, as outlined in each figure legend. The horizontal line corresponds to an NAT concentration of 20 µM. (**A**) displays a bi-plot of NAT with phenylalanine, where the hashed vertical line represents 140 µM phenylalanine. (**B**) displays a bi-plot of NAT with leucine, where the hashed vertical line represents 290 µM leucine. PN+: administered total parenteral nutrition; Normal: presumptive normals (*n* = 120); PN+NegElv: PN+ with no elevated biomarkers (*n* = 42); PN+PosElv: PN+ with one or more elevated biomarkers (*n* = 80); TYRSN1: neonate with tyrosinemia type I (TYRSN1, OMIM 276700, *n* = 1).

Figure 3A,B display bi-plots with NAT and amino acids that were acquired by FIA-MS/MS on a triple quadrupole platform. Figure 3A displays NAT and Phe concentrations of residual NBS specimens, along with hashed lines denoting 20 µM NAT and 140 µM Phe (i.e., 2023 average U.S. Phe cutoff). In the lower left quadrant, one can see the coalescence of presumptive normals, PN+ specimens with no elevations in biomarkers (PN+NegElv), a few PN+PosElv specimens with NAT < 20 µM, and a TYRSN1 confirmed case. The upper left quadrant had only PN+ specimens, while the upper right quadrant had only PN+PosElv specimens. In the lower right quadrant, one can see the four PN+PosElv specimens that had NAT < 20 µM and Phe concentrations exceeding 140 µM. Figure 3B displays NAT and Leu concentrations of the residual NBS specimens, along with hashed lines denoting 20 µM NAT and 290 µM Leu (i.e., 2023 average U.S. Leu cutoff). The distribution of data in Figure 3B’s bi-plot is similar to Figure 3A. In Figure 3A,B, one can see that several of the seven PN+PosElv specimens with NAT < 20 µM showed elevated concentrations of amino acids, which we feel supports our hypothesis that six of the seven PN+PosElv specimens without NAT (Figure 1B,C) were removed from PN prior to DBS collection. Some NICUs discontinue PN administration to normalize amino acids while continuing glucose administration several hours before DBS collection to minimize NBS inconclusive specimens [26]. It is also important to note that in Figure 3A,B, the two specimens with the highest NAT had the highest levels of each amino acid.

**Figure 4:**
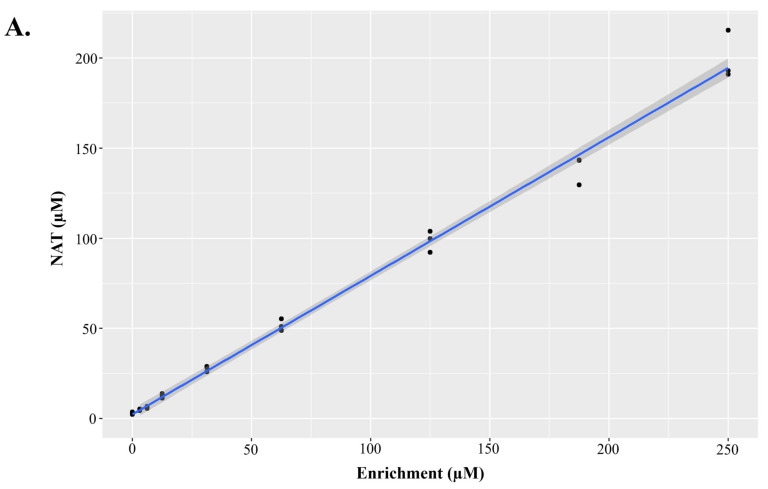

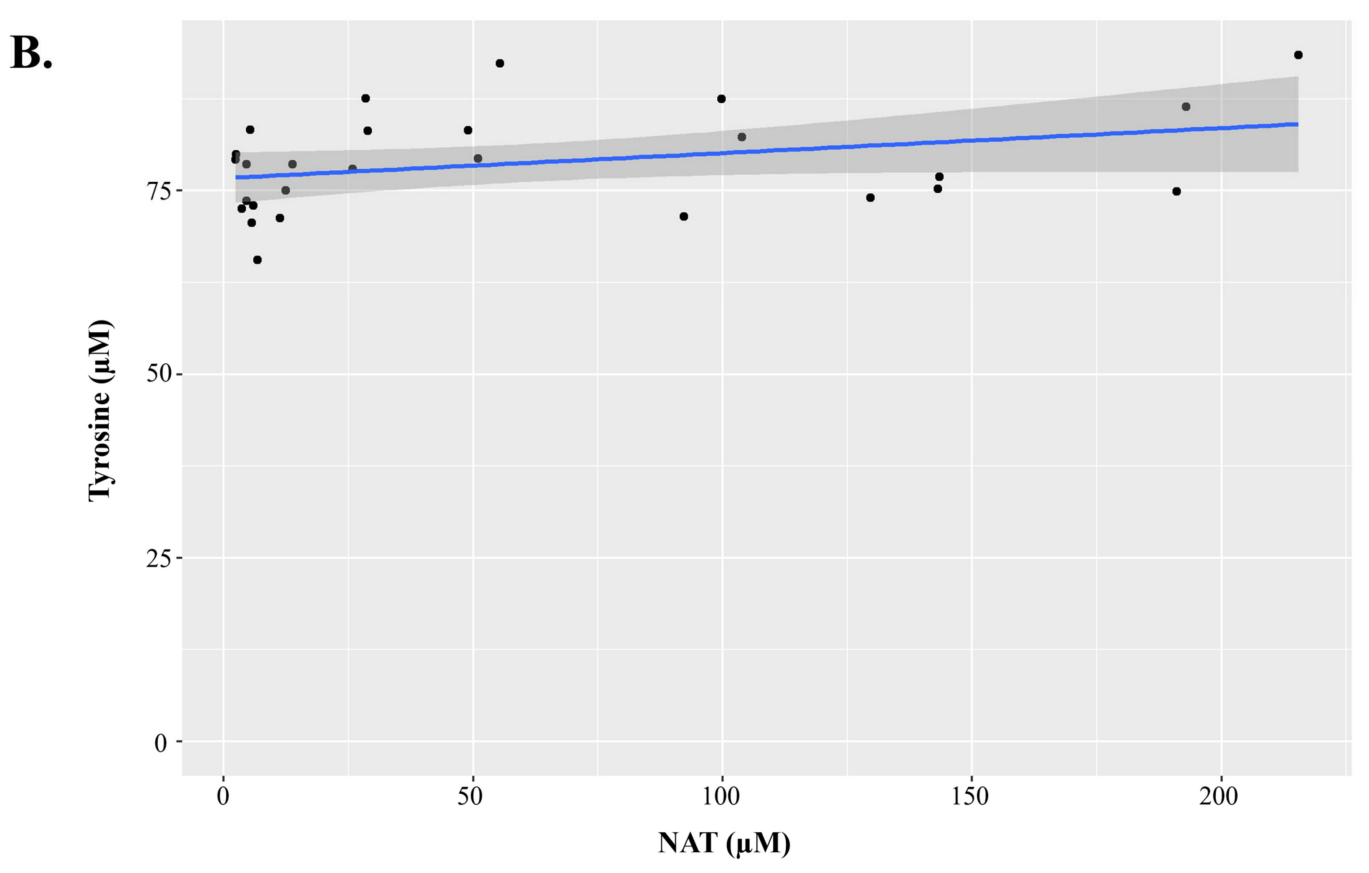
Characteristics of dried blood spot materials containing N-acetyltyrosine (NAT) for method validation. In order to validate the method containing NAT as a biomarker, NAT dried blood spot materials were created for method validation. NAT was linearly enriched into whole blood, then spotted onto dried blood spot cards. (**A**) displays a bi-plot, with measured NAT concentration on the y-axis and the enriched NAT concentration on the x-axis, with an overlayed regression line. The slope of the regression line was 0.77, with an R^2^ of 0.99. (**B**) demonstrates that in these validation materials, NAT did not hydrolyze to tyrosine. The measured concentration of tyrosine is presented on the y-axis and the measured concentration of NAT on the x-axis, with an overlayed regression line. The slope of the regression line was 0.034, with an R^2^ of 0.08.

NAT quantification in our cohort of residual NBS specimens demonstrates positive clinical utility, so we validated the multiplexed method. It is important to note that in addition to NAT and NAT IS, we added direct ISs for C5:1, C10:0, and C14:1, which have historically used surrogate ISs in NBS assays. Furthermore, we created nine-level DBS NAT linearity materials, spanning from 0 to 250 µM, for use in validation (Figure 4) since none of the QC and linearity DBS materials currently manufactured by our group contain NAT. Figure 4A demonstrates that the NAT response was linear across the enriched concentrations, while Figure 4B demonstrates that NAT did not hydrolyze to Tyr in these validation materials (i.e., NAT was stable in the DBS material). The analytical validation parameters presented included precision, linearity, and the estimated limit of detection (LOD) and limit of quantification (LOQ) using the Taylor method [30], which are presented in Table 1. The concentrations of DBS QC and linearity materials are presented in Tables S1 and S2. Precision was measured by analyzing low, middle, and high DBS QCs in triplicate on 20 separate days, so the data are presented as the precision range across the three QC levels. Precision was ≤~16% for all biomarkers (Table 1). Precision data on each of the QC pools individually are presented in Table S4. Linearity was assessed by analyzing the manufactured linearity materials in triplicate on a single day, using the measured and expected concentration of each biomarker. All biomarkers analyzed in Table 1 had R^2^ values ≥ 0.99. Estimated LODs and LOQs were sufficient for most biomarkers, and we calculated the signal-to-noise ratio for several biomarkers that had higher-than-expected estimated LODs and LOQs, which are presented in Table S5. Since many NBS labs are already screening Tyr using IS Tyr-^13^C_6_, we also calculated NAT precision using this surrogate IS, and these similar results are presented in Table S6.

**Figure 5:**
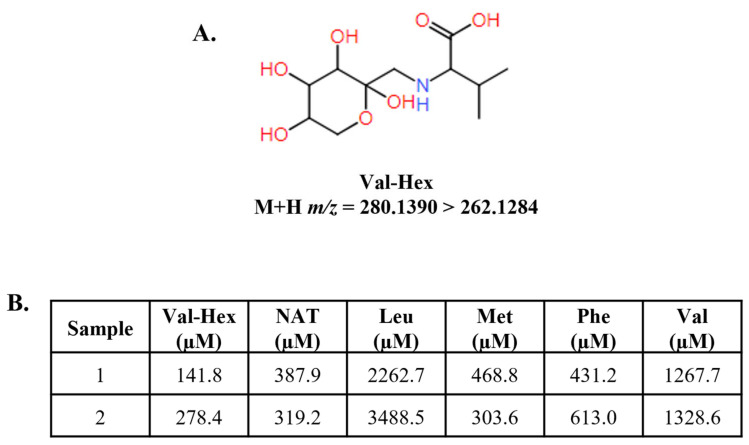
Structure, transition, and concentration of the Amadori rearrangement production (ARP) valine–hexose (Val-Hex). (**A**) displays the structure of the ARP Val-Hex, along with the M+H parent and product ion *m*/*z* presented as a transition. We hypothesized that ARPs would form since the parenteral nutrition administered would contain high dextrose and amino acids. Val-Hex was the most abundant ARP in our sample cohort. (**B**) displays the Val-Hex, NAT, and several other amino acids for two PN+PosElv neonates. These two neonates had unusual amino acid profiles that appeared similar to an improper specimen collection (i.e., IV port collection instead of a heel prick collection). Val-^2^H_8_ was used as a surrogate internal standard for Val-Hex quantification. Val-Hex data were quantified by high-resolution mass spectrometry, while other biomarkers were analyzed by triple quadrupole mass spectrometry. Figure S3A–F highlight these two neonates in the histogram of each biomarker in (**B**). PN+PosElv: PN+ with one or more elevated biomarkers; Leu: leucine; Met: methionine; NAT: N-acetyltyrosine; Phe: phenylalanine.

Lastly, we hypothesized that since dextrose and amino acids are simultaneously present during PN administration, amino acid-dextrose conjugates called Amadori rearrangement products (ARPs) would be detectable. Recently, ARPs were demonstrated as biomarkers for some IEMs [31], and we included ARP transitions during the HRMS reanalysis of residual NBS specimens. Unfortunately, the major product ion of ARPs is intramolecular dehydration, which is a very common small molecule dissociation product, so to add confidence to our identification, data were acquired using a mass resolving power of 60,000, and we used the accurate product ion *m*/*z* with a ∆ 5-ppm threshold as integration criteria. Val–hexose (Val-Hex) was present in 57.5% of PN+PosElv specimens compared to 22.5% Phe–hexose, 3.75% Met–hexose, 0% Tyr–hexose, and 0% Leu–hexose. The structure of Val-Hex is presented in Figure 5A, along with the M+H *m*/*z* of the parent and product ion. More information regarding the mass spectra and confirmation of Val-Hex identification is presented in Figure S3. Since we found no internal standards commercially available for ARPs, we used Val-^2^H_8_ as a surrogate IS for Val-Hex quantification.

Val-Hex was undetectable in over 98% of normals, the average Val-Hex concentration was 0.465 µM, and it was undetectable in the TYRSN1 specimen. The presence of elevated Val-Hex was unique to neonates with reported PN administration, especially in the PN+PosElv group, which appears to indicate elevations may be associated with higher concentrations of PN components. As mentioned, the two neonates with the highest NAT also had the highest levels of Phe and Leu (Figure 3A,B). Figure 5B displays the amino acid profiles for these two neonates, which had Val-Hex concentrations exceeding 100 µM. Figure S4A displays the histogram of Val-Hex concentrations, and no other PN+ neonate had Val-Hex > 50 µM. Furthermore, the two neonates with Val-Hex > 100 µM had the highest level of nearly every amino acid (Figure S4A–F). The amino acid profiles resembled specimens where the clinical staff collected DBSs from PN IV ports, which contaminates the blood with the PN solution and is considered improper DBS specimen collection. The CLSI guidelines state that DBSs should be collected by heel prick, and while not documented in the literature, it is known by NBS laboratories and clinical follow-up teams that improper specimen collection does sometimes occur. Taken together, we hypothesize that Val-Hex, NAT, and other amino acids could be used as a screening algorithm for improper DBS collection during PN administration.

## 4. Discussion

In our current study, we hypothesized the presence of NAT in neonatal DBSs could be utilized as a biomarker of PN administration. Using residual NBS DBS specimens, we confirmed NAT was present in 74 of the 80 PN+PosElv specimens and hypothesized the remaining six were transitioned to enteral feeding or fasted hours prior to DBS collection, thus giving sufficient time for NAT to be excreted in the urine. Roughly 50% of PN+NegElv specimens had NAT present in the blood. The remainder likely had DBS collection before or shortly after PN administration (i.e., there was not enough time for NAT and amino acids to be elevated in the blood prior to collection). NAT was not detected in any presumptive normals nor was it present in the TYRSN1 specimen. There are currently two U.S. NBS laboratories actively piloting NAT and Val-Hex screening in a clinical setting. Manning et al. recently presented their preliminary NAT clinical results at the 2023 Association for Public Health Laboratories NBS Symposium [32]. We are working with three additional U.S. NBS laboratories to aid the pilot adoption of NAT and Val-Hex into their assays.

The biomarkers NAT and Val-Hex address several challenges associated with previously reported PN administration markers. In 2010, we identified unique *m*/*z* signals from elevated dextrose in PN solutions using butyl ester analysis of DBSs from PN+ samples [33]. However, the use of butyl ester derivatization in NBS has steadily declined over the past decade [34], and dextrose is undetectable in DBSs without derivatization. Val-Hex may allow the detection of PN-contaminated blood for both amino acids and dextrose in non-butyl ester assays. Other potential PN biomarkers, like transthyretin [35] and phthalates [36], have been suggested, but proteins are precipitated during NBS small molecule extraction, and phthalates in DBS may not be detectable under FIA-MS/MS conditions. Val-Hex and NAT offer the added benefit of being easily integrated into existing NBS methods using surrogate ISs Val-^2^H_8_ and Tyr-^13^C_6_, respectively.

NAT detection in blood after administration of NAT-containing PN solutions has been well-known for several decades [20]. However, to our knowledge, our study is the first to hypothesize and demonstrate the clinical utility of NAT as a PN administration biomarker in first-tier NBS assays. In neonates and children < 12 months, the poor metabolism of NAT was suggested as unique since the phenomenon was not observed in children aged one to seven [23]. In 2001, glycyl-L-tyrosine was demonstrated to be a more bioavailable source of Tyr in neonates, with extremely low, if any, excretion of the dipeptide [14]. While we mention the association between low thyroxine and low Tyr as possibly being related to Tyr deficiency during PN administration [15], it was suggested that newborns administered PN for more than 15 days could suffer from iodine deficiency, resulting in low thyroxine levels [37]; however, most neonates are transferred to enteral nutrition sooner to support the growth and development of the GI system [38]. While sources of Tyr and PN solution composition were outside the scope of our study, it is possible the immature metabolic pathways of premature neonates could explain the low Tyr when administered PN. If manufacturers alter PN formulas to contain more bioavailable forms of Tyr in the future, it should be considered to have some NAT present as it provides NBS laboratories the ability to determine PN status and blood contaminated with PN.

We acknowledge that NAT’s rapid urinary excretion limits its utility, making it detectable in blood only during PN administration and for a few hours after PN is discontinued, and overall, there is limited information regarding NAT as a metabolite in humans. While we state NAT is not an endogenous metabolite in the blood, it is important to note NAT has been found in the urine of TYRSN1, tyrosyluria [20,21], and tyrosinosis [39]. Therefore, we acknowledge that NAT is produced in humans through a mechanism historically associated with clearing excess Tyr. More recently, in 2020, NAT was demonstrated in stressed animal models to function as an intrinsic factor of mitohormesis [40]. Also, this study reported NAT detection in what appeared to be 80 µL pooled adult human serum [40], and a comparison of their spectral results, the pure NAT standard (Figure S1C), and the spectral results from our PN+ specimens (Figure S1B) are all in agreement. We acknowledge it is possible NAT could be a low-abundant circulating metabolite in adults that we were not able to detect in 3.1 µL of whole blood on DBSs collected from neonates shortly after birth. It is also important to note that NAT is sold as a nutritional supplement, similar to N-acetylcysteine. Therefore, while very unlikely in our opinion, it may be possible that a mother consuming an NAT dietary supplement shortly before giving birth or while breastfeeding could result in NAT elevations in their newborn’s DBS; however, we found no data in the literature to support this.

Our study confirms NAT as a reliable biomarker of PN administration. Since there is an interferent in the NAT transition, differences in extraction solvent composition, buffers, and mass spectrometer source and conditions are expected to influence observed NAT concentrations and cutoffs. Therefore, NBS laboratories may obtain different quantitative values for NAT compared to our study. Using our assay and instrument, an NAT concentration ≥ 20 µM indicates PN use, with positive correlations observed between NAT levels and other elevated analytes during PN administration. However, NAT concentrations < 20 µM in PN-annotated samples did not always correspond with normal levels of other analytes. This discrepancy is likely due to NAT’s rapid urinary excretion (within 4–8 h) [20], while amino acids and C5 can take up to 48 h to normalize [41]. The sample cohort size may be considered a limitation of our study, but since our laboratory does not perform routine NBS, we rely on receiving residual NBS specimens from U.S. public health NBS laboratories. These labs only know if PN was administered around the time of sample collection, without details on the type of PN or whether the newborn was fasting. Some hospitals practice glucose-only PN or fasting before sample collection to minimize false biomarker elevations and reduce the need for repeat tests [26]. Further studies should include detailed data on feeding and PN doses within the 6 h period before collection.

Once the clinical utility of our assays was demonstrated and disseminated, our group transferred the technology to NBS laboratories for pilot testing in thousands of samples for clinical validation. Based on early disclosures and discussions with various stakeholders, along with our recent disclosure of these study results at the 2023 Association for Public Health Laboratories NBS Symposium [32], we anticipate the adoption of NAT as a biomarker of PN administration. To support adoption, starting in 2024, we began piloting the addition of NAT into DBS-based quality assurance materials. We are currently working to obtain freshly made PN solutions from a hospital to confirm our Val-Hex hypothesis. Future directions should include using NAT in combination with other biomarkers to investigate the possibility of developing algorithms that can aid the identification of premature neonates with IEMs being administered PN to enhance diagnosis and accelerate treatment initiation. In conclusion, the addition of NAT in first-tier NBS assays provides the ability to identify PN+ specimens that may be incorrectly annotated, along with other potential uses in clinical chemistry and neonatology.

## Figures and Tables

**Table 1:** Table of precision ranges, linearity parameters, and estimated limits of detection and quantification.

Biomarker	Precision Range	R^2^	Estimated LOD	Estimated LOQ
Alanine	[6.56–8.86]	0.99	23.5	78.2
Arginine	[13.71–15.32]	0.99	1.6	5.2
C0	[6.23–8.47]	0.99	2.4	8.0
C2	[6.69–9.17]	0.99	3.2	11
C3	[6.62–8.87]	0.99	0.37	0.99
C3DC + C4OH	[7.62–10.15]	0.99	0.16	0.2
C4	[6.39–9.8]	0.99	0.04	0.1
C5	[5.97–8.47]	0.99	0.07	0.2
C5:1	[6.87–10.62]	0.99	0.04	0.1
C5DC	[8.23–14.19]	0.98	0.2	0.5
C5OH	[6.3–8.42]	0.99	0.3	0.9
C6	[7.39–9.17]	0.99	0.05	0.2
C8	[8.24–9]	0.99	0.3	1.0
C10	[7.52–13.59]	0.99	0.1	0.4
C10:1	-	0.99	0.07	0.3
C10:2	-	0.99	0.07	0.2
C12	[7.06–9.68]	0.99	0.1	0.4
C14:0	[6.31–8.56]	0.99	0.1	0.3
C14:1	[6.02–8.14]	0.99	0.1	0.3
C16	[6.86–8.62]	0.99	0.4	1.2
C16OH	[8.66–9.84]	0.99	0.06	0.2
C18:0	[6.79–8.41]	0.99	0.3	0.9
C18:1	-	0.99	0.4	1
C18OH	[14.51–15.34]	0.99	0.09	0.3
Citrulline	[12.84–13.52]	0.99	15.0	49.8
Creatine	[5.05–6.82]	0.99	13.6	45.4
Creatinine	[5.84–9.97]	0.99	2.7	9.1
Glycine	[6.83–8.86]	0.99	26.8	89.3
GUAC	[8.68–12.11]	0.99	0.9	2.9
Leucine	[5.87–7.51]	0.99	4.6	15.4
Methionine	[13.96–14.48]	0.99	2.8	9.4
NAT	[8.99–14.59]	0.99	3.2 *	10.7 *
Ornithine	[14.01–15.65]	0.99	10.4	34.6
Phenylalanine	[5.88–8.8]	0.99	5.5	18.4
SUAC	[7.95–12.02]	0.99	0.3	0.9
Tyrosine	[6.95–9.68]	0.99	6.6	21.9
Valine	[6.19–9.21]	0.99	4.6	15.4

Precision data are presented as the [minimum to maximum] residual standard deviation, expressed as a percentage, calculated across three quality-control dried blood spot pools. Precision was assessed by analyzing each of the three quality control pools in duplicate on twenty separate days. Linearity was assessed by analyzing dried blood spot linearity pools in triplicate on a single day. The R^2^ was calculated by regressing the measured concentration of each analyte on the expected value of each analyte. Limits of detection (LODs) and limits of quantification (LOQs) were estimated using the Taylor method [30]. Three dried blood spot pools from linearity materials were analyzed in triplicate on seven separate days. The standard deviation was regressed on the mean of these data to generate regression parameters. The LOD was calculated by multiplying the intercept (S0) by 3, and the LOQ was calculated by multiplying the intercept by 10. The asterisk (*) denotes that this biomarker had a negative intercept, and for this biomarker, the standard deviation of the lowest linearity pool was assumed as the intercept (S0) to estimate the LOD and LOQ, as described above. Since the Taylor method is an estimation of LOD and LOQ, we calculated the signal-to-noise in the base QC pools for several analytes that have higher estimated LOD and LOQ (Table S5).

## Data Availability

The data presented in this study are available on request from the corresponding author.

## Supplementary Materials

The following supporting information can be downloaded at: https://www.mdpi.com/article/10.3390/ijns10040081/s1, Table S1: Linearity material certification data; Table S2: Mean and enriched concentration from QC certification data; Table S3: List of metabolite name, m/z, and internal standards; Table S4: Method validation precision results; Table S5: Signal-to-noise calculation on select biomarkers; Table S6: Comparison of N-acetyltyrosine method precision using direct internal standard and surrogate tyrosine internal standard; Figure S1: Identification of N-acetyltyrosine interference; Figure S2: Bubble correlogram of biomarker values from neonates administered with parenteral nutrition; Figure S3: Identification of valine-hexose by high-resolution mass spectrometry; Figure S4: Histograms of select biomarkers in only neonates administered parenteral nutrition, with denotation of two neonates with suspected improper specimen collection. References cited in Supplementary Materials [42,43].

## Abbreviations

PN	Parenteral nutrition
IV	Intravenous
TPN	Total parenteral nutrition
NICU	Neonatal intensive care unit
Phe	Phenylalanine
Tyr	Tyrosine
NAT	N-acetyltyrosine
NBS	Newborn screening
IEMs	Inborn error of metabolism
FIA-MS/MS	Flow injection analysis tandem mass spectrometry
Met	Methionine
QC	Quality control
ISs	Internal standard
PN+	Neonate with reported parenteral nutrition administration
PN+PosElv	Neonate with reported parenteral nutrition administration and elevations in one or more biomarkers
HRMS	High-resolution mass spectrometry
Arg	Arginine
Leu	Leucine
C5	Isovalerylcarnitine
Val	Valine
PN+NegElv	Neonate with reported parenteral nutrition administration and no elevations in biomarkers
TYRSN1	Tyrosinemia type 1
LOD	Limit of detection
LOQ	Limit of quantification
ARP	Amadori rearrangement product
Val-Hex	Valine–hexose
