# Supplementary material for: N-Acetyltyrosine as a Biomarker of Parenteral Nutrition Administration in First-Tier Newborn Screening Assays
Source: Int J Neonatal Screen. 2024 Dec 10;10(4):81. doi: 10.3390/ijns10040081 (PMC11678415; doi:10.3390/ijns10040081)
Supplement: Supplementary file 1 [file IJNS-10-00081-s001.zip › IJNS-3306819-supplementary.pdf]

Supplementary Materials

S1. Supplementary Methods

S1.1. Sample Type and Preparation

Appropriate safety control measures (including engineering, administrative policy and procedure, and personal protective equipment) were used for all procedures based on a site-specific risk assessment that identified physical, health, and procedural hazards. The Centers for Disease Control and Prevention's (CDC) Newborn Screening Quality Assurance Program (NSQAP) quality control (QC) and linearity dried blood spots (DBS) cards were used in method development. All blood products used in method development and analytical validation were acquired from a commercial source and did not meet the definition of human subjects as specified in 45-Code of Federal Regulations (CFR) 46.102 (f). The analysis of de-identified residual newborn specimens at the CDC laboratory was determined not to constitute engagement in human subject research.

The specimens used in this study included MSMS1QC (Lot# A-D2215) and linearity materials (Lot#20211-9) containing amino acids and acylcarnitines, and validation linearity materials enriched with only N-acetyltyrosine (NAT), since NAT is not yet enriched along with amino acids and acylcarnintines in our current manufactured materials. QC DBS cards included a base pool and three enriched versions of the base pool, at varying enrichment levels for each metabolite, and QC production was performed as previously described [1]. MSMS1and NAT linearity DBS cards included a nine-level set of materials. The DBS cards were prepared by serially diluting a highly enriched blood pool with non-enriched blood. Additional information regarding pool preparation and concentrations can be found in the **Table S1** (i.e., linearity) and Quality Control Specimen Certification Data are presented in **Table S2**. Deidentified residual clinical newborn specimens were provided by the Texas Department of State Health Services Newborn Screening Laboratory, and the authors did not have access to any information on these individuals other than whether they were classified as presumptive normals (n=120), neonates administered PN that had no elevated biomarkers (PN+NegElv, n=42), neonates administered PN that had one or more elevated biomarker (PN+PosElv, n=80), and Tyrosinemia type I (TYRSN1, OMIM 276700, n=1). Clinical specimens were randomized prior to sample extraction and analysis.

S1.2. Chemical, Standard, and Consumable Information

Optima LC/MS Acetonitrile, Optima LC/MS Water (Fisher Scientific, Waltham, MA, USA), and LC/MS grade formic acid (Thermo Scientific GmbH, Bremen, Germany) were used in extraction, resuspension, and the mobile phase. All samples were extracted, processed, and resuspended in Corning 3365 96-well round bottom polypropylene plates (VMR International Corp, Suwanee, GA, USA). The internal standards used for quantification are as follows: NSK-A [Lot# PR-33246], NSK B-1 [Lot# PR-34365], NSK-B-G1-1 [Lot# PR-33902], creatine [methyl-D_3, Lot# PR-30122], creatinine [N-methyl-D_3, Lot# I-23033], succinylacetone [3,4,5,6,7-$^{13}C_5$, 99%, Lot# PR-32732], guanidinoacetic acid [1,2-$^{13}C_2$ 97-99%, $^{15}N_3$ 97-99%, 97% pure, Lot# PR-32405], adenosine [$^{13}C_5$, 97%, Lot# I-22540], and deoxyadenosine [$^{13}C_5$, 98%, Lot# I-27685], tiglylcarnitine [N-methyl-D_9, 98%, Lot# PR-33096], decanoylcarnitine [N-methyl-D_3, 98%, Lot# PR-23942], tetradecenoylcarnitine [N-methyl-D_9, 98%, Lot# PR-33097], NAT [$^{13}C_6$, 97%, Lot# PR-33482] (Cambridge Isotope Laboratories Inc., Andover, MA, USA), and hydroxystearoylcarnitine [N-methyl-D_3, 99%, Lot#BCCG3211] (MilliporeSigma, St. Louis, MO, USA).

S1.3. Sample Extraction

The same extraction protocol was used for preparing first-tier and second-tier QC and linearity specimens, along with residual clinical newborn specimens. Samples were extracted and analyzed as previously described [2], but modified as specified. Extraction working IS (WISS) solution was comprised of 80/20 acetonitrile/water, 0.05% formic acid,

and 0.015% hydrazine hydrate. 100 μL of WISS were added to each DBS punch, then subjected to heated extraction for 45 min at 45 ^{0}C. DBS extracts were transferred to clean wells, the plate was sealed, then transferred to the autosampler for FIA-MS/MS analysis.

S1.4. Instrumentation

Data were acquired by FIA using an Acquity liquid chromatography system coupled to a Waters Xevo TQD tandem mass spectrometer (MS/MS, Milford, MA, USA). High resolution mass spectrometry (HRMS) analysis of samples was performed using a Q-Exactive HF HRMS (Thermo Scientific GmbH, Bremen, Germany), coupled to an Agilent 1290 Infinity LC (Agilent Technologies, Santa Clara, CA, USA). An isocratic mobile phase was used on both instruments composed of 50% acetonitrile, 49.98% water, 0.02% formic acid.

S1.5. Peak Integration and Quantification

Peak integration was performed using the target analyte and IS SRM data for each pair using the Waters Neolynx software. Quantification was performed using the integrated peak areas (PA) so that concentration was calculated as follows, where 3.1 μL is the volume of blood in a 3.2 mm DBS punch.

$$[Analyte]_{Blood} = [IS]_{Extract} \times \frac{PA_{Analyte}}{PA_{IS}} \times \frac{V_{Extract}}{V_{Blood}} = [IS]_{Extract} \times \frac{PA_{Analyte}}{PA_{IS}} \times \frac{100\ \mu L}{3.1\ \mu L}$$

Waters ESI source parameters

**Parameter**	**Value**
Capillary kV	3
Desolvation Temp ^{0}C	250
Desolvation (L/hr)	400
Cone (L/hr)	12
Source Temp ^{0}C	150

Waters inlet parameters

**Time (min)**	**Flow (mL/min)**
Initial	0.150
0.1	0.150
0.2	0.020
0.7	0.600
0.9	0.150

Inlet mobile phase was 100% A, using a curve setting of 11.

Table S1. Linearity material certification data.

	Linearity Pools								
**Biomarker**	**1**	**2**	**3**	**4**	**5**	**6**	**7**	**8**	**9**
Alanine	188.10	200.60	213.10	238.10	313.10	438.10	688.10	938.10	1188.10
Arginine	5.78	10.15	14.53	23.28	49.53	93.28	180.78	268.28	355.78
C0	15.00	21.25	27.50	40.00	77.50	140.00	265.00	390.00	515.00
C2	10.80	13.92	17.05	23.30	42.05	73.30	135.80	198.30	260.80
C3	0.92	1.55	2.17	3.42	7.17	13.42	25.92	38.42	50.92
C3DC+C4OH	0.05	0.30	0.55	1.05	2.55	5.05	10.05	15.05	20.05
C4	0.12	0.24	0.37	0.62	1.37	2.62	5.12	7.62	10.12
C5	0.07	0.32	0.57	1.07	2.57	5.07	10.07	15.07	20.07
C5:1	0.00	0.13	0.25	0.50	1.25	2.50	5.00	7.50	10.00
C5DC	0.03	0.16	0.28	0.53	1.28	2.53	5.03	7.53	10.03

C5OH	0.78	1.03	1.28	1.78	3.28	5.78	10.78	15.78	20.78
C6	0.02	0.15	0.27	0.52	1.27	2.52	5.02	7.52	10.02
C8	0.02	0.52	1.02	2.02	5.02	10.02	20.02	30.02	40.02
C10	0.08	0.33	0.58	1.08	2.58	5.08	10.08	15.08	20.08
C10:1	0.01	0.26	0.51	1.01	2.51	5.01	10.01	15.01	20.01
C10:2	0.00	0.25	0.50	1.00	2.50	5.00	10.00	15.00	20.00
C12	0.02	0.27	0.52	1.02	2.52	5.02	10.02	15.02	20.02
C14:0	0.06	0.31	0.56	1.06	2.56	5.06	10.06	15.06	20.06
C14:1	0.02	0.27	0.52	1.02	2.52	5.02	10.02	15.02	20.02
C16	0.75	1.25	1.75	2.75	5.75	10.75	20.75	30.75	40.75
C16OH	0.01	0.14	0.26	0.51	1.26	2.51	5.01	7.51	10.01
C18:0	0.57	0.82	1.07	1.57	3.07	5.57	10.57	15.57	20.57
C18:1	1.07	1.32	1.57	2.07	3.57	6.07	11.07	16.07	21.07
C18OH	0.01	0.13	0.26	0.51	1.26	2.51	5.01	7.51	10.01
Citrulline	14.61	33.36	52.11	89.61	202.11	389.61	764.61	1139.61	1514.61
Creatine	184.87	194.87	204.87	224.87	284.87	384.87	584.87	784.87	984.87
Creatinine	24.07	29.07	34.07	44.07	74.07	124.07	224.07	324.07	424.07
Glycine	197.93	216.68	235.43	272.93	385.43	572.93	947.93	1322.93	1697.93
GUAC	1.33	1.83	2.33	3.33	6.33	11.33	21.33	31.33	41.33
Leucine	34.52	59.52	84.52	134.52	284.52	534.52	1034.52	1534.52	2034.52
Methionine	3.43	9.68	15.93	28.43	65.93	128.43	253.43	378.43	503.43
‡NAT	2.84	5.96	9.09	15.34	34.09	65.34	127.84	190.34	252.84
Ornithine	47.08	53.33	59.58	72.08	109.58	172.08	297.08	422.08	547.08
Phenylalanine	15.49	40.49	65.49	115.49	265.49	515.49	1015.49	1515.49	2015.49
SUAC	0.22	1.47	2.72	5.22	12.72	25.22	50.22	75.22	100.22
Tyrosine	20.15	38.90	57.65	95.15	207.65	395.15	770.15	1145.15	1520.15
Valine	36.92	49.42	61.92	86.92	161.92	286.92	536.92	786.92	1036.92

Data are presented as the expected value (i.e., concentration of biomarker in unenriched pool plus concentration of enriched biomarker in each pool) were disseminated from 20211-9 linearity material certification reports and were analyzed as non-derivatized sample extracts by FIA-MS/MS. Concentrations are presented as µM. ‡ denotes data reported from these biomarkers are from NAT validation materials and are reported as the expected value.

Table S2. Mean and enriched concentration from QC certification data.

Biomarker	Base	Low	Medium	High
Alanine	220 (0)	496.6 (200)	648.4 (400)	791.3 (600)
Arginine	9.1 (0)	90.5 (100)	170.1 (200)	241 (300)
C0	13.14 (0)	26.19 (10)	38.07 (20)	51.3 (30)
C2	6.66 (0)	16.82 (10)	26.65 (20)	37.07 (30)
C3	0.74 (0)	4.31 (4)	7.83 (8)	11.42 (12)
C3DC+C4OH	0.03 (0)	0.26 (1)	0.53 (2.5)	1.22 (5.5)
C4	0.07 (0)	0.77 (1)	2.21 (3)	3.67 (5)
C5	0.05 (0)	0.56 (0.5)	1.58 (1.5)	3.08 (3)
C5:1	0 (0)	0.29 (0.5)	0.87 (1.5)	1.72 (3)
C5DC	0.03 (0)	0.53 (0.5)	1.05 (1)	2.49 (2.5)
C5OH	0.62 (0)	1.6 (1)	2.48 (2)	3.44 (3)
C6	0.01 (0)	0.44 (0.5)	0.87 (1)	2.16 (2.5)
C8	0.02 (0)	0.5 (0.5)	0.98 (1)	2.38 (2.5)
C10	0.11 (0)	0.54 (0.5)	1.01 (1)	2.48 (2.5)
C12	0.01 (0)	0.91 (1)	1.85 (2)	2.72 (3)
C14:0	0.04 (0)	0.47 (0.5)	1.39 (1.5)	2.73 (3)
C14:1	0.01 (0)	0.21 (0.5)	0.68 (1.5)	1.35 (3)
C16	0.62 (0)	3.63 (4)	6.67 (8)	9.71 (12)
C16OH	0.01 (0)	0.22 (0.25)	0.86 (1)	1.25 (1.5)
C18:0	0.46 (0)	1.23 (1)	2.81 (3)	4.28 (5)
C18OH	0 (0)	0.13 (0.25)	0.53 (1)	0.77 (1.5)
Citrulline	13.1 (0)	37.4 (25)	103.9 (100)	227.5 (250)

Creatine	181.1 (0)	227.4 (50)	357.7 (200)	520.9 (400)
Creatinine	17.4 (0)	59.3 (50)	99.7 (100)	179.5 (200)
Glycine	223.2 (0)	474.1 (300)	677.6 (600)	867.8 (900)
GUAC	0.9 (0)	4.6 (5)	8.5 (10)	16 (20)
Leucine	70 (0)	226.2 (150)	327.5 (300)	560.2 (600)
Methionine	8.6 (0)	9.4 (12.5)	48.4 (62.5)	155.4 (187.5)
‡NAT	2.8 (0)	12.48 (12.5)	51.8 (62.5)	143.3 (187.5)
Ornithine	143 (0)	267.2 (100)	343 (200)	404.8 (300)
Phenylalanine	27.9 (0)	175.7 (150)	297.3 (300)	429 (450)
SUAC	0.22 (0)	1.25 (2.5)	4.32 (10)	7.7 (20)
Tyrosine	25.3 (0)	273 (300)	515.7 (600)	750.7 (900)
Valine	70 (0)	267.3 (200)	378.5 (350)	508.3 (500)

Data are presented as the mean characterized value of each pool with the pool enrichment value in parentheses. Data were disseminated from 2215 QC material certification reports and were analyzed as non-derivatized sample extracts by FIA-MS/MS. Concentrations are presented as µM. ‡ denotes data reported from these biomarkers are from NAT validation materials. Base corresponds to NAT linearity pool 1, low corresponds to NAT pool 2, medium corresponds to NAT pool 6, and high corresponds to NAT pool 8 in **Table S1**.

Table S3. List of metabolite name, *m/z*, and internal standards.

Biomarker (Short Name)	Parent ion (*m/z*)	Product Ion (*m/z*)	Cone (V)	Collison Energy (eV)	Internal Standard
Glycine (Gly)	76.0	30.0	20	10	Gly-$^{13}C_2{}^{15}N$
Glycine (Gly-$^{13}C^{15}N$)	78.0	32.0	20	10	
Alanine (Ala)	90.0	44.0	20	11	Ala-D_3
Alanine (Ala-D_3)	94.0	48.0	20	11	
Creatinine (Crn)	114.1	44.0	32	18	Crn-D_3
Creatinine-$^2H_3$ (Crn-D_3)	117.1	47.0	32	18	
Guanidinoacetic acid (GUAC)	118.0	76.0	30	14	GUAC-$^{13}C_2$-$^{15}N_1$
Valine (Val)	118.05	72.05	24	14	Val-D_8
Guanidinoacetic acid-$^{13}C_2{}^{15}N$ (GUAC-$^{13}C_2{}^{15}N$)	121.0	79.0	30	14	
Valine-$^2H_8$ (Val-D_8)	126.05	80.05	24	14	
Creatine (Cre)	132.1	90.1	25	16	Crn-D_3
Leucine (Leu)	132.05	86.05	22	13	Leu-D_3
Ornithine (Orn)	133.1	70.0	23	20	Orn-D_2
Creatine-$^2H_3$ (Cre-D_3)	135.1	93.1	25	16	
Ornithine-$^2H_2$ (Orn-D_2)	135.1	72.0	23	20	
Leucine-$^2H_3$ (Leu-D_3)	135.05	89.05	22	13	
Methionine (Met)	150.05	104.0	23	14	Met-D_3
Methionine-$^2H_3$ (Met-D_3)	153.05	107.0	23	14	
Succinylacetone-hydrazone (SUAC)	155.05	137.1	23	15	SUAC-$^{13}C_5$
SUAC-$^{13}C_5$-hydrazone (SUAC-$^{13}C_5$)	160.05	142.1	23	15	
Free Carnitine (C0)	162.1	103.0	37	24	C0-D_9
Free Carnitine-$^2H_3$ (C0-D_3)	165.1	103.0	37	24	C0-D_9
Phenylalanine (Phe)	166.1	120.1	25	16	Phe-$^{13}C_6$
Free Carnitine-$^2H_9$ (C0-D_9)	171.1	103.0	37	24	
Phenylalanine-$^{13}C_6$ (Phe-$^{13}C_6$)	172.1	126.1	25	16	
Arginine (Arg)	175.1	70.0	28	26	Arg-^{13}C-D_4
Citrulline (Cit)	176.1	113.0	23	22	Cit-D_2
Citrulline-$^2H_2$ (Cit-D_2)	178.1	115.0	23	22	
Arginine-$^{13}C^2H_4$ (Arg-$^{13}CD_4$)	180.1	75.0	28	26	
Tyrosine (Tyr)	182.1	136.0	25	17	Tyr-$^{13}C_6$
Tyrosine-$^{13}C_6$ (Tyr-$^{13}C_6$)	188.1	142.0	25	17	
Acetylcarnitine (C2)	204.1	85.0	33	26	C2-D_3
Acetylcarnitine-$^2H_3$ (C2-D_3)	207.1	85.0	33	26	
Propionylcarnitine (C3)	218.1	85.0	35	28	C3-D_3

Propionylcarnitine-$^{2}H_3$ (C3-D_3)	221.1	85.0	35	28	
N-acetyltyrosine (NAT)	224.1	136.1	22	17	NAT-$^{13}C_6$
N-acetyltyrosine-$^{13}C_6$ (NAT-$^{13}C_6$)	230.1	142.1	22	17	
Butyrylcarnitine (C4)	232.1	85.0	35	30	C4-D_3
Butyrylcarnitine-$^{2}H_3$ (C4-D_3)	235.1	85.0	35	30	
Tiglylcarnitine (C5:1)	244.2	85.0	35	32	C5:1-D_9
Isovalerylcarnitine (C5:0)	246.2	85.0	35	30	C5-D_9
Malonylcarnitine (C3DC)	248.1	85.0	35	30	C4-D_3
Hydroxybutyrylcarnitine (C4OH)	248.1	85.0	35	30	C4-D_3
Deoxyadenosine (dADO)	252.15	136.1	35	20	dADO-$^{13}C_5$
Tiglylcarnitine-$^{2}H_9$ (C5:1-D_9)	263.1	85.0	35	32	
Isovalerylcarnitine-$^{2}H_9$ (C5-D_9)	255.2	85.0	35	30	
Deoxyadenosine-$^{13}C_5$ (dADO-$^{13}C_5$)	257.15	136.1	35	20	
Hexanoylcarnitine (C6)	260.2	85.0	35	30	C5-D_9
Hydroxyisovalerylcarnitine (C5OH)	262.2	85.0	35	30	C5OH-D_3
Hydroxyisovalerylcarnitine-$^{2}H_3$ (C5OH-D_3)	265.2	85.0	35	30	
Adenosine (ADO)	268.15	136.1	35	24	ADO-$^{13}C_5$
Adenosine-$^{13}C_5$ (ADO-$^{13}C_5$)	273.15	136.1	35	24	
Glutarylcarnitine (C5DC)	276.2	85.0	38	34	C5DC-D_3
Glutarylcarnitine-$^{2}H_3$ (C5DC-D_3)	279.2	85.0	38	34	
Octanoylcarnitine (C8)	288.2	85.0	38	33	C8-D_3
Octanoylcarnitine-$^{2}H_3$ (C8-D_3)	291.2	85.0	38	33	
Decadienoylcarnitine (C10:2)	312.2	85.0	40	36	C10:0-D_3
Decenoylcarnitine (C10:1)	314.2	85.0	40	36	C10:0-D_3
Decanoylcarnitine (C10:0)	316.2	85.0	40	36	C10:0-D_3
Decanoylcarnitine-$^{2}H_3$ (C10:0-D_3)	319.3	85.0	40	36	
Dodecanoylcarnitine (C12:0)	344.3	85.0	48	40	C12-D_9
Dodecanoylcarnitine-$^{2}H_9$ (C12-D_9)	353.3	85.0	48	40	
Tetradecenoylcarnitine (C14:1)	370.3	85.0	52	43	C14:1-D_9
Tetradecanoylcarnitine (C14:0)	372.3	85.0	50	43	C14:0-D_9
Tetradecenoylcarnitine-$^{2}H_9$ (C14:1-D_9)	379.3	85.0	52	43	
Tetradecanoylcarnitine-$^{2}H_9$ (C14:0-D_9)	381.3	85.0	50	43	
Palmitoylcarnitine (C16)	400.4	85.0	53	44	C16-D_3
Palmitoylcarnitine-$^{2}H_3$ (C16-D_3)	403.4	85.0	53	44	
Hydroxyhexadecanoylcarnitine (C16OH)	416.4	85.0	55	44	C16OH-D_3
Hydroxyhexadecanoylcarnitine-$^{2}H_3$ (C16OH-D_3)	419.4	85.0	55	44	
Oleoylcarnitine (C18:1)	426.4	85.0	54	45	C18:0-D_3
Stearoylcarnitine (C18:0)	428.4	85.0	54	45	C18:0-D_3
Stearoylcarnitine-$^{2}H_3$ (C18:0-D_3)	431.4	85.0	54	45	
Hydroxystearoylcarnitine (C18OH)	444.4	85.0	54	35	C18OH-D_3
Hydroxystearoylcarnitine-$^{2}H_3$ (C18OH-D_3)	447.1	85.0	54	35	

Complete list of amino acid and acylcarnitines name, analyte short names, parent *m/z* and corresponding product ion, and internal standard used for quantitation.

Table S4. Method validation precision results.

Biomarker	Low QC	Medium QC	High QC
Alanine	361.47 ± 23.71 (6.56%)	514.08 ± 40.95 (7.97%)	655.57 ± 58.08 (8.86%)
Arginine	59.76 ± 8.47 (14.18%)	112.96 ± 15.49 (13.71%)	170.33 ± 26.1 (15.32%)
C0	14.25 ± 0.89 (6.23%)	20.25 ± 1.51 (7.45%)	27.09 ± 2.29 (8.47%)
C2	14.61 ± 0.98 (6.69%)	21.66 ± 1.92 (8.89%)	30.05 ± 2.76 (9.17%)
C3	3.65 ± 0.24 (6.62%)	6.2 ± 0.48 (7.71%)	8.9 ± 0.79 (8.87%)
C3DC+C4OH	0.32 ± 0.02 (7.62%)	0.62 ± 0.06 (9.77%)	1.46 ± 0.15 (10.15%)
C4	0.65 ± 0.04 (6.39%)	1.61 ± 0.14 (8.87%)	2.84 ± 0.28 (9.8%)
C5	0.41 ± 0.02 (5.97%)	1.09 ± 0.08 (7.76%)	2.21 ± 0.19 (8.47%)
C5:1	0.29 ± 0.03 (10.62%)	0.84 ± 0.06 (6.87%)	1.71 ± 0.12 (7.17%)
C5DC	0.47 ± 0.07 (14.19%)	0.94 ± 0.08 (8.23%)	2.3 ± 0.24 (10.61%)

C5OH	1.06 ± 0.07 (6.3%)	1.6 ± 0.13 (8.32%)	2.22 ± 0.19 (8.42%)
C6	0.32 ± 0.02 (7.39%)	0.6 ± 0.06 (9.17%)	1.59 ± 0.14 (9.12%)
C8	0.42 ± 0.04 (8.79%)	0.77 ± 0.07 (9%)	1.98 ± 0.16 (8.24%)
C10	0.4 ± 0.05 (13.59%)	0.62 ± 0.07 (10.77%)	1.51 ± 0.11 (7.52%)
C12	0.64 ± 0.05 (7.06%)	1.23 ± 0.12 (9.68%)	1.94 ± 0.17 (9.04%)
C14:0	0.45 ± 0.03 (6.31%)	1.23 ± 0.1 (8.11%)	2.54 ± 0.22 (8.56%)
C14:1	0.24 ± 0.01 (6.02%)	0.64 ± 0.05 (8.14%)	1.37 ± 0.11 (8.06%)
C16	3.18 ± 0.22 (6.86%)	5.78 ± 0.42 (7.34%)	8.68 ± 0.75 (8.62%)
C16OH	0.23 ± 0.02 (9.68%)	0.84 ± 0.07 (8.66%)	1.29 ± 0.13 (9.84%)
C18:0	1.12 ± 0.09 (7.6%)	2.55 ± 0.17 (6.79%)	4 ± 0.34 (8.41%)
C18OH	0.26 ± 0.04 (14.51%)	1.04 ± 0.16 (15.34%)	1.63 ± 0.24 (14.88%)
Citrulline	28.01 ± 3.79 (13.52%)	76.82 ± 9.86 (12.84%)	181.22 ± 23.86 (13.17%)
Creatine	191.57 ± 9.67 (5.05%)	279.04 ± 17.41 (6.24%)	407.77 ± 27.8 (6.82%)
Creatinine	60.43 ± 3.53 (5.84%)	101.96 ± 8.45 (8.28%)	195.64 ± 19.5 (9.97%)
Glycine	365.18 ± 24.95 (6.83%)	547.85 ± 39.23 (7.16%)	727.42 ± 64.42 (8.86%)
GUAC	4.63 ± 0.56 (12.11%)	7.88 ± 0.81 (10.27%)	15.31 ± 1.33 (8.68%)
Leucine	185.46 ± 10.89 (5.87%)	285.07 ± 21.17 (7.43%)	525.76 ± 39.46 (7.51%)
Methionine	41.3 ± 5.77 (13.96%)	109.38 ± 15.83 (14.48%)	185.24 ± 26.77 (14.45%)
NAT	14.96 ± 2.18 (14.59%)	51.21 ± 6.09 (11.88%)	170.58 ± 15.34 (8.99%)
Ornithine	134.25 ± 20.84 (15.52%)	189.07 ± 26.5 (14.01%)	252.07 ± 39.44 (15.65%)
Phenylalanine	149.84 ± 8.81 (5.88%)	260.53 ± 20.01 (7.68%)	389.62 ± 34.27 (8.8%)
SUAC	0.97 ± 0.12 (12.02%)	4.01 ± 0.39 (9.82%)	7.11 ± 0.57 (7.95%)
Tyrosine	249.13 ± 17.31 (6.95%)	466.52 ± 42.36 (9.08%)	700.08 ± 67.8 (9.68%)
Valine	189.5 ± 11.73 (6.19%)	289.13 ± 21.53 (7.45%)	397.15 ± 36.57 (9.21%)

Validation of method precision was performed by taking duplicate measurements of the three QC pools on 20 separate days. Data are presented as average ± standard deviation [percent residual standard deviation (RSD%)].

Table S5. Signal-to-noise calculation on select biomarkers.

**Biomarker**	**Concentration**	**Signal-to-Noise**
Arginine	4.29	7812
C3DC+C4OH	0.05	3588
C5	0.05	5917
C5:1	0.1	109904
C5OH	0.47	21464
C5DC	0.02	750
C6	0.01	1569
C8	0.01	1600
C10	0.12	15964
C10:1	0.17	308288
C10:2	0.13	267840
C12	0.01	1304
C14:1	0.03	3413
C14	0.03	4799
C16	0.53	88112
C16OH	0.02	3508
C18:0	0.45	82720
C18:1	0.61	115280
C18OH	0.01	2137
Citrulline	7.82	1491
GUAC	1.26	828
NAT	3.66	2168
SUAC	0.77	92000

Since the estimated limit of detection using the Taylor method was high for several biomarkers, we calculated the signal-to-noise using Waters Mass Lynx software for the

lowest enriched concentration of each biomarker from extracted DBS. These data demonstrated the validated method has significant analytical sensitivity for biomarkers commonly screened by newborn screening laboratories.

Table S6. Comparison of N-acetyltyrosine method precision using direct internal standard and surrogate tyrosine internal standard.

Biomarker	Low QC	Medium QC	High QC
NAT / NAT-$^{13}C_6$	14.96 ± 2.18 (14.59%)	51.21 ± 6.09 (11.88%)	170.58 ± 15.34 (8.99%)
NAT / Tyr -$^{13}C_6$	15.20 ± 2.51 (16.50%)	49.5 ± 5.58 (11.30%)	141.0 ± 23.0 (16.30%)

NAT/Tyr -$^{13}C_6$ were calculated to demonstrate the use of a surrogate internal standard for NAT quantification was possible, since Tyr -$^{13}C_6$ internal standard is present in mass spectrometry NBS assays. It is important to note the collision energies were different for NAT and Tyr -$^{13}C_6$, so it is possible that precision results would improve if collision energies matched for surrogate internal standard quantification. Method precision data were calculated using the formula presented in the *Peak integration and quantification* section above, using peak areas from method validation. The NAT / NAT-$^{13}C_6$ data are identical to **Table S4** and are displayed for comparison purposes. Data are presented as average ± standard deviation [percent residual standard deviation (RSD%)]. NAT: N-acetyltyrosine; Tyr: tyrosine.

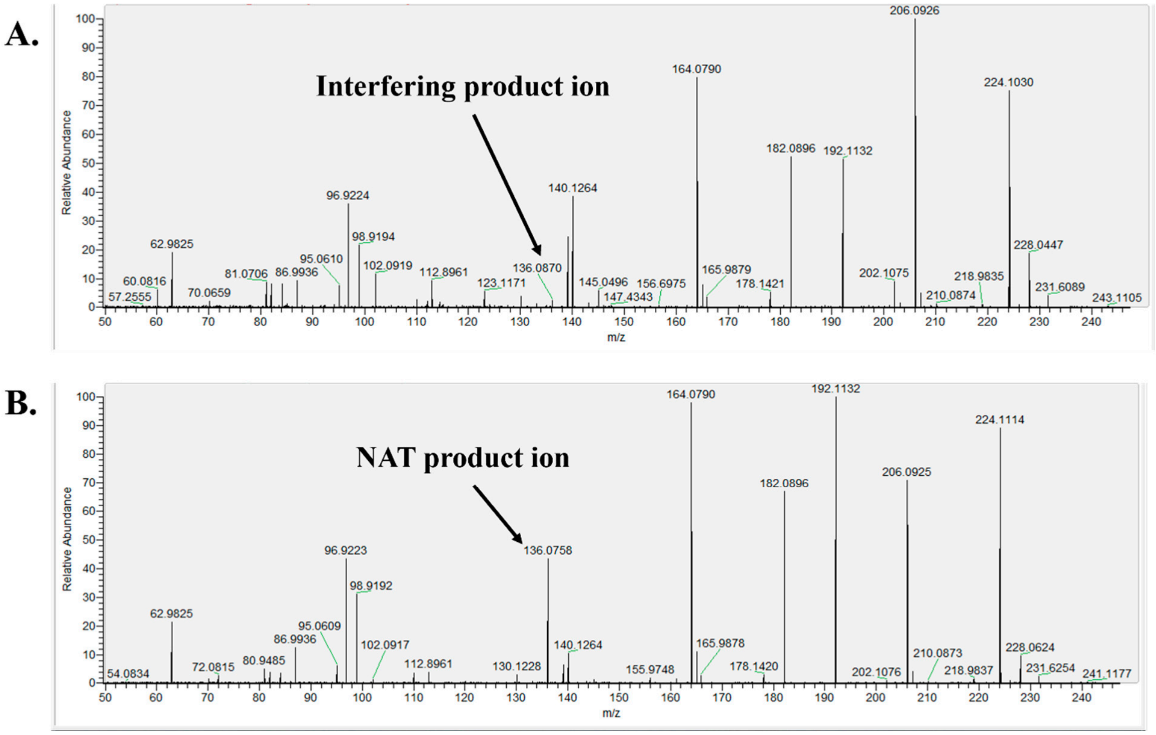

Figure S1. Identification of N-acetyltyrosine interference (**A**,**B**).

Preliminary data indicated higher than expected concentrations of NAT under FIA-MS/MS conditions on nominal mass spectrometers. We hypothesized that other small molecules in the matrix shared a transition interfering with the *m/z* 224.1 > 136.1 quantification of NAT. **Figure S1A** displays a *m/z* 224.1 HRMS parallel reaction monitoring spectra (PRM) of a presumptive normal residual clinical sample extracted with WISS. **Figure S1B** displays a *m/z* 224.1 HRMS PRM of a PN+PosElv residual clinical sample extracted with WISS. The product ion matches the observed product ion from a neat NAT standard, which is shown in **Manuscript Figure 1**.

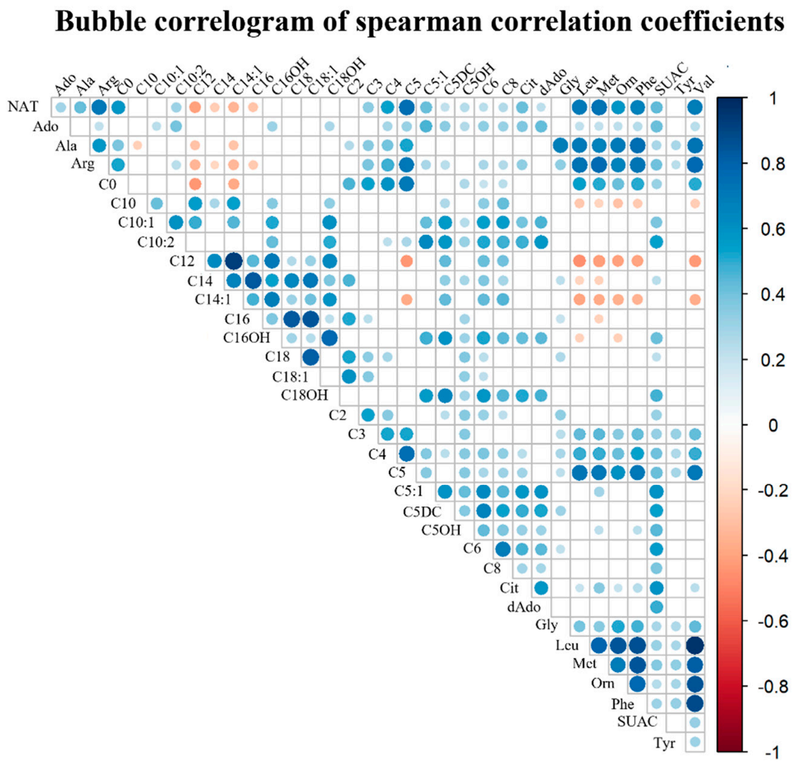

Figure S2. Bubble correlogram of biomarker values from neonates administered with parenteral nutrition.

Bubble correlogram using data from neonates administered parenteral nutrition (PN+, n=122). Spearman correlations were performed and coefficients were plotted using a bubble correlogram, where the size of the bubble and color denote the direction of correlation and proximity to 1 or -1.

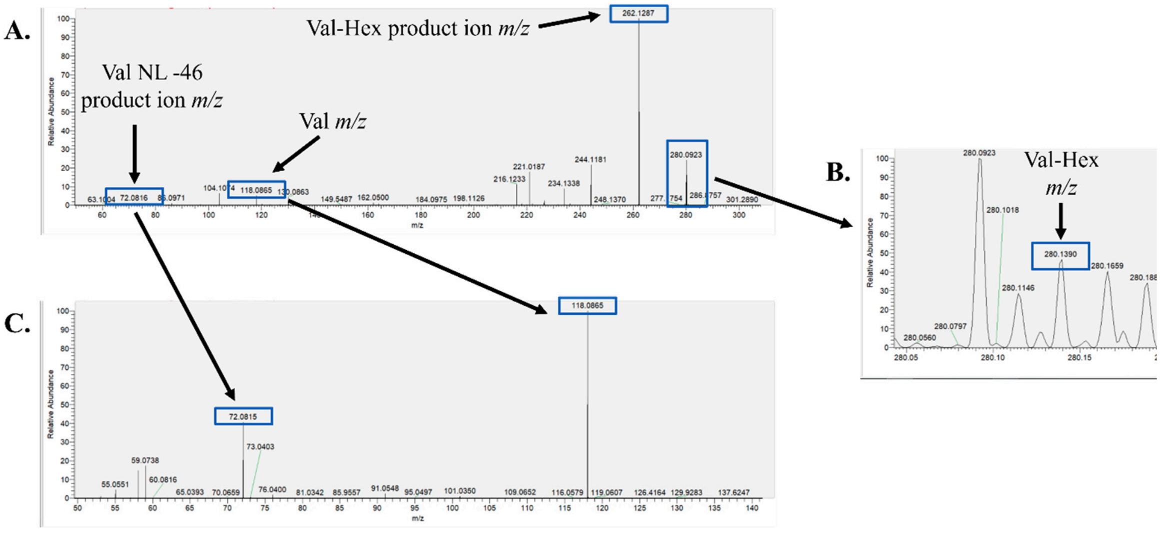

Figure S3. Identification of valine-hexose by high-resolution mass spectrometry (**A**–**C**).

Valine-hexose (Val-Hex) structure was confirmed using HRMS. **Figure S3A** displays the PRM spectra obtained from DBS extracts of the newborn with the highest Val-Hex and NAT. The dehydration product ion of Val-Hex theoretical *m/z* is 262.1284 and the observed

was *m/z* 282.1287 (Δ 1.14 ppm mass error). The valine parent ion is also present which has a Δ -2.63 ppm mass error from the theoretical *m/z* of 118.0868. **Figure S3B** displays a HRMS selected ion monitoring scan from this specimen highlighting the accurate *m/z* of Val-Hex with a Δ 0 ppm mass error. **Figure S3C** displays a HRMS PRM of pure valine, which can be compared to the PRM spectra of **Figure S3A**. Taken together, our identification of Val-Hex is based on the accurate parent *m/z* of Val-Hex, loss of water product ion *m/z*, valine parent ion *m/z*, and valine product ion *m/z*, all within the expected mass error.

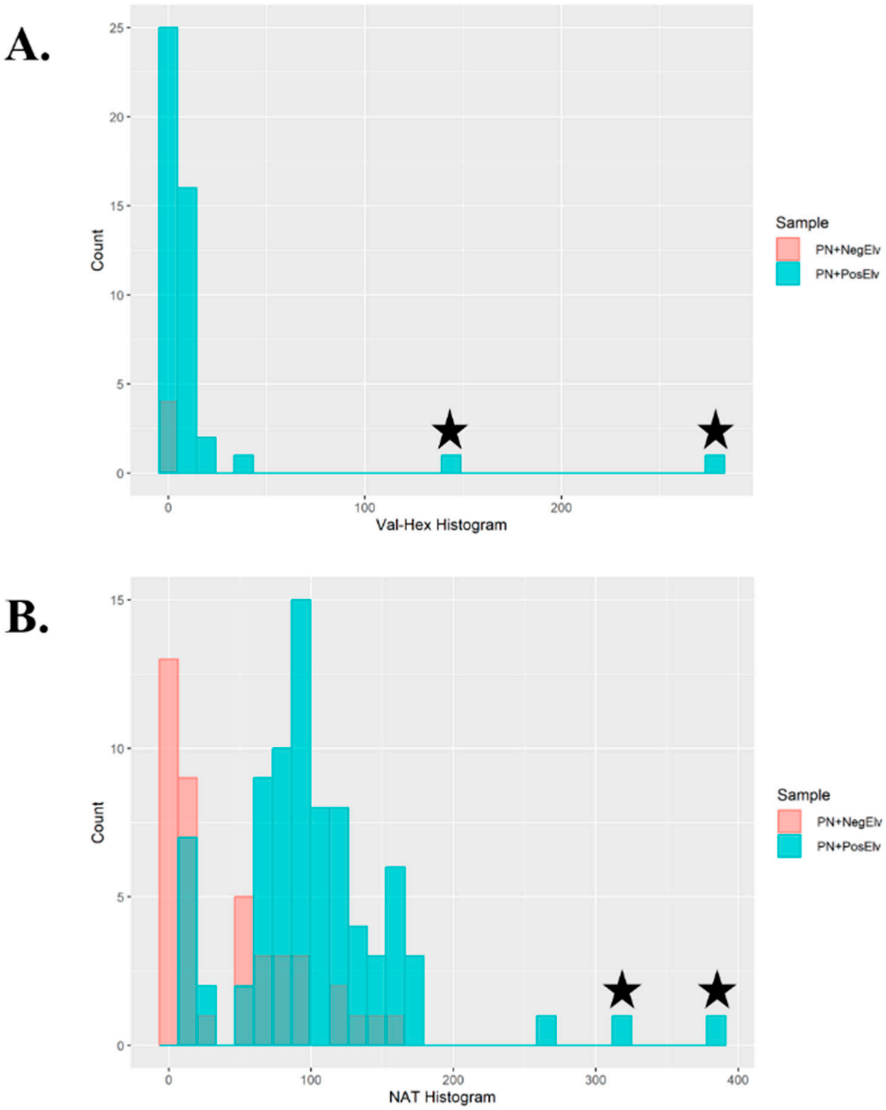

C.

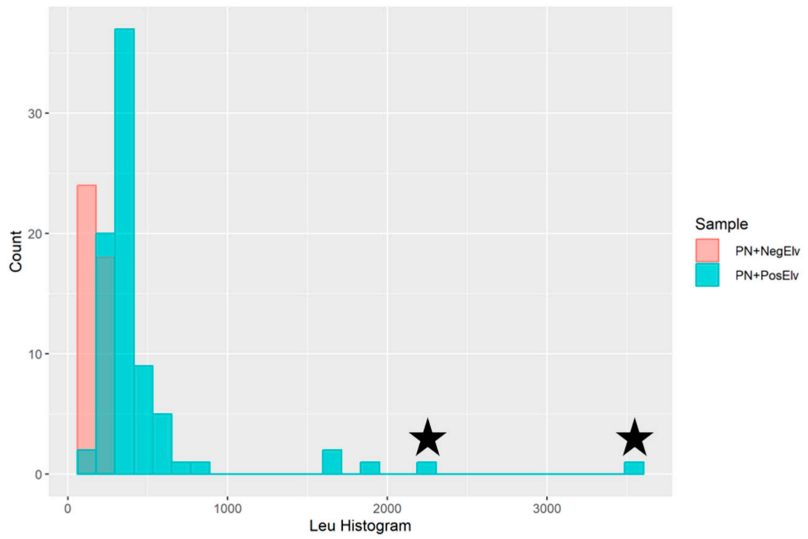

Count
0
10
20
30
0
1000
2000
3000
Leu Histogram
Sample
PN+NegElv
PN+PosElv

D.

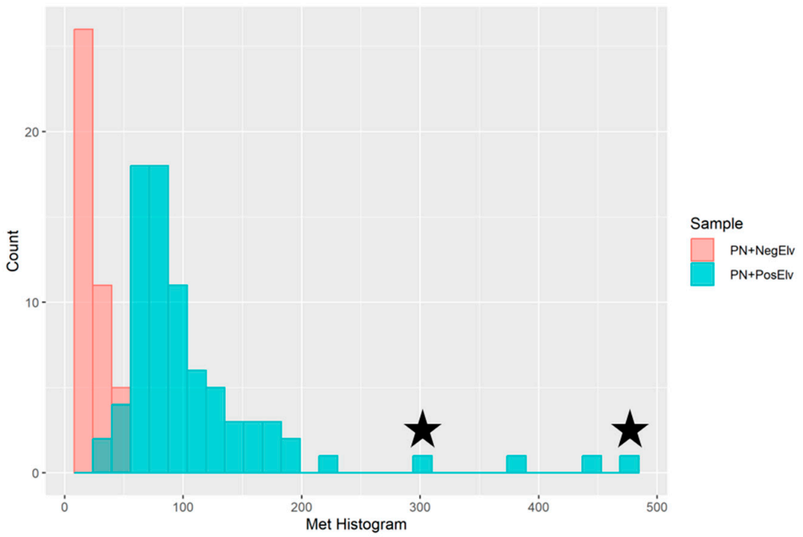

Count
0
10
20
0
100
200
300
400
500
Met Histogram
Sample
PN+NegElv
PN+PosElv

E.

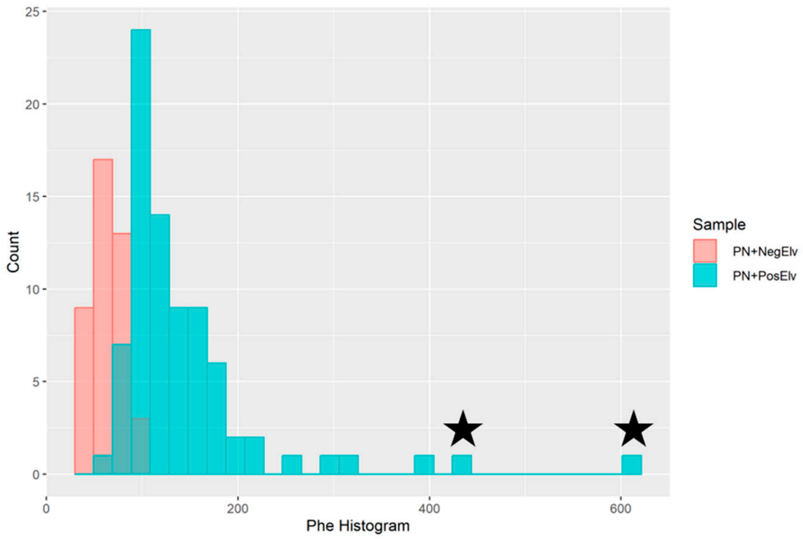

Count
0
5
10
15
20
25
0
200
400
600
Phe Histogram
Sample
PN+NegElv
PN+PosElv

F.

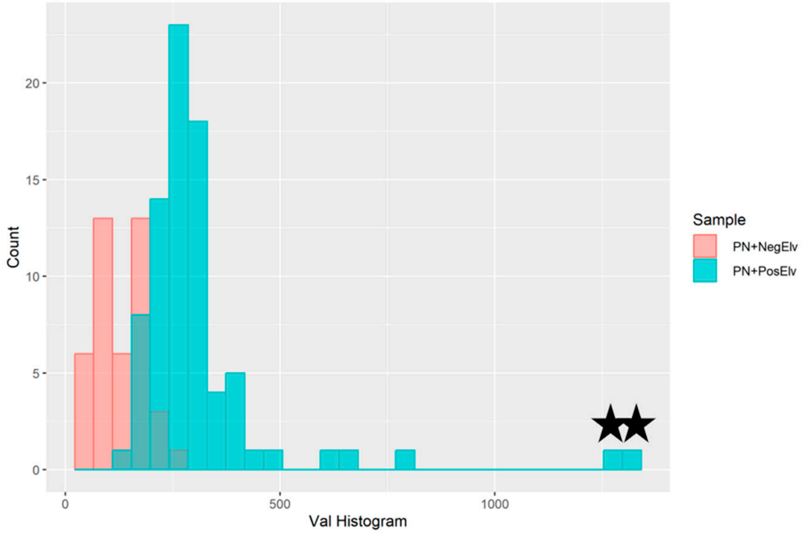

Figure S4. Histograms of select biomarkers in only neonates administered parenteral nutrition, with denotation of two neonates with suspected improper specimen collection (**A**–**F**).

Figure S4 displays histograms of select biomarkers for PN+ specimens with the two highest NAT and Val-Hex specimens denoted with black stars. These two specimens were hypothesized to have improper DBS specimen collection.

References

1. De Jesús, V.R.; Mei, J.V.; Cordovado, S.K.; Cuthbert, C.D. The Newborn Screening Quality Assurance Program at the Centers for Disease Control and Prevention: Thirty-five Year Experience Assuring Newborn Screening Laboratory Quality. *Int. J. Neonatal Screen* **2015**, *1*, 13–26.
2. Young, B.; Hendricks, J.; Foreman, D.; Pickens, C.A.; Hovell, C.; De Jesús, V.R.; Haynes, C.; Petritis, K. Development of dried blood spot quality control materials for adenosine deaminase severe combined immunodeficiency and LC-MS/MS method for their characterization. *Clin. Mass Spectrom.* **2020**, *17*, 4–11.
